# The Relation between Pregnancy-Associated Plasma Protein A and Obstructive Sleep Apnea Syndrome

**DOI:** 10.1155/2018/3297810

**Published:** 2018-06-03

**Authors:** Ali Cengiz, Suat Konuk, Tuncer Tuğ

**Affiliations:** ^1^Pulmonology Department, Ağrı State Hospital, Ağrı, Turkey; ^2^Pulmonology Department, Faculty of Medicine, Abant Izzet Baysal University, Bolu, Turkey

## Abstract

**Aim:**

We aimed to investigate the relationship between serum pregnancy-associated plasma protein A (PAPP-A) levels and obstructive sleep apnea syndrome (OSAS).

**Materials and Method:**

44 patients with OSAS and 44 healthy adults were included in this study. The participants having rheumatic or systemic inflammatory disease, advanced liver or kidney failure, diabetes, heart failure, hypertension, pregnancy, prerenal azotemia, known history of coronary artery disease, any pulmonary disease, rhinitis, or atopy, history of major trauma or surgery within the last six 6 months, and inhaled nasal or systemic corticosteroid use or other anti-inflammatory medications and those with <18 years of age were excluded. Serum PAPP-A levels were determined by the Elisa method with the immune sandwich measuring method. Statistical analysis of the study was performed with SPSS 17.0 statistical analysis package program, and *p* < 0.05 was considered as significant.

**Results:**

Serum PAPP-A levels of patients with OSAS (2.350 ng/ml (0.641–4.796)) were significantly higher (*p* < 0.001) when compared with healthy controls (0.971 ng/ml (0.109–2.679)). There was a statistically significant difference in serum PAPP-A levels between groups of OSAS patients according to the classification of OSAS severity. Between the groups of patients with OSAS, serum levels of PAPP-A in moderate group was significantly higher when compared with severe OSAS group (*p* < 0.001). There was positive correlations between PAPP-A levels and night minimum (*p*=0.042, *r*=0.309), and average oxygen levels (*p*=0.006, *r*=0.407). There was a negative correlation between PAPP-A levels and AHI (*p*=0.002, *r*=−0.460).

**Conclusion:**

Higher PAPP-A levels in OSAS patients that were found in this study show inflammatory component in OSAS.

## 1. Introduction

Obstructive sleep apnea syndrome (OSAS) is characterized by permanent recurrent obstruction of the upper airways during sleep. Obstructive apnea is the respiratory effort in response to a totally or partially obstructed airway. Apnea results in loud snoring or arousal when the airway passage is enable again [1, 2]. OSAS is seen in approximately 1–5% of adult men and 1.2–2.5% of women [3–8].

Although the pathogenesis of OSAS could not exactly be clarified, there are studies reporting that chronic inflammation plays a role. It is asserted that recurrent hypoxia attacks in OSAS cause the release of various inflammatory mediators and the initiation of inflammatory process. Chronic inflammation, vasoconstriction as a result of endothelial dysfunction and vascular damage, and hypercoagulability-thrombotic tendency occur. In this case, it leads to cardiovascular and cerebrovascular complications in OSAS patients. In people with OSAS, soluble adhesion molecules like intercellular adhesion molecule 1 (ICAM-1), vascular adhesion molecule 1 (VCAM-1), and acute phase proteins like C-reactive protein (CRP), interleukin-6 (IL-6), retinol binding protein (RBP), and isoprostane are reported to be high [9, 10]. It is known that many factors such as advanced age, male sex, obesity, medications, genetic factors, airway configuration and diameter, sleep position, upper airway muscle and reflexes, and cytokines contribute to the development of OSAS [11, 12].

Pregnancy-associated plasma protein A (PAPP-A) was first defined by Lin et al. in 1974 [13]. PAPP-A is a high molecule weighted zinc binding proatherosclerotic metalloproteinase [14, 15]. PAPP-A is first synthesized by placenta trophoblasts during pregnancy. It is used for Down Syndrome screening in pregnancy [16]. In addition to trophoblasts, PAPP-A is reported to be synthesized in endometrium, testicle, kidney, bone, colon and other adult and fetal tissues [15]. It is reported that PAPP-A is an inflammatory marker and detected in high levels in patients with asthma, allergic rhinitis, lung cancer, and chronic obstructive pulmonary disease (COPD) [15, 17–19]. In some studies, a relation between insulin-like growth factor 1 (IGF-1) and PAPP-A levels in patients with acute coronary syndrome (ACS) was revealed. It was asserted that PAPP-A may be a new acute coronary syndrome indicator showing the atherosclerotic plaque instability [16, 20].

In this study, we aimed to investigate the relation of PAPP-A levels and OSAS.

## 2. Materials and Methods

This study was conducted at the Abant İzzet Baysal University, Department of Chest Diseases, Sleep Disorders Center, between December 2015 and July 2016. The ethical committee approval was obtained from the corporate ethical committee (no. 2016/07). Written informed consent was obtained from all participants.

### 2.1. Patients and Controls

Forty-four patients subjected to a polysomnographic test at the sleep laboratory for OSAS prediagnosis and 44 healthy controls were included to the study. The inclusion criteria for OSAS patients were determined as apnea-hypopnea index (AHI) higher than 5, no previous treatments for OSAS, no other systemic diseases, no major surgical interventions or trauma during last 6 months, no administration of inhaled or systemic medications, and age above 18 years. Healthy controls were chosen among the individuals who were relatives or friends of the hospital staff.

### 2.2. Polysomnography

Patients were evaluated using Epworth Sleepiness Scale (ESS) and polysomnography (PSG) that was applied all night (Grass Technologies Comet Series EEG/PSG with AS40 Amplifier System). The Epworth Sleepiness Scale (ESS) is a quantitative method to evaluate sleepiness or the propensity to fall asleep in eight everyday life situations, through a Likert scale [21–23]. This instrument has been used in different contexts, populations, and clinical conditions, with particular interest in respiratory sleep disorders [21, 24].

As standard measurement parameters, three-channel electroencephalogram (EEG) (F4-M1, C4-M1, O4-M1), two-channel electrooculogram (EOG), electromyogram (EMG) (jaw, right, and left tibialis anterior), body position, oronasal thermal sensors, nasal pressure sensor, thoracoabdominal respiration movements, electrocardiography (ECG), respiratory sound recording, oxygen saturation measurements from the fingertip, and synchronous video recording were taken. Abnormal respiration events related to sleep were scored manually according to American Association of Sleep Medicine (AASM, 2012) criteria.

Obstructive sleep apnea was defined as decrease in airflow by more than 90% or total interruption for at least 10 seconds while chest and abdominal wall movements continued. Hypopnea was defined as at least 30% decrease in oronasal airflow for 10 seconds or more together with at least 3% oxygen desaturation compared to the level before respiratory event or the development of arousal. AHI was calculated by dividing the sum of obstructive apnea and hypopnea to total sleep duration. Independent from the symptoms, those with AHI >15 were immediately accepted to have OSAS. Besides, subjects who have any one of apnea and/or snoring, excessive daytime sleepiness, fatigue, insomnia, or nonrestorative sleep symptoms together with AHI >5 were also accepted to have OSAS.

Patients with OSAS were evaluated in three groups as mild (5–15), moderate (16–30), and severe (>30) OSAS in terms of AHI and in two groups as low (<10) and high (≥10) according to Epworth score.

### 2.3. Pulmonary Function Tests

Pulmonary function tests (PFT) was conducted with Vmax Sensormedics 229 spirometry device. After the subjects performed three acceptable maneuvers, the best pulmonary function test result was recorded. The exhaling activity was observed to last at least 6 seconds. At the end of the activity, FEV1, FVC, FEV1/FVC, FEF 25–75, and PEF values of the patients were measured and recorded.

### 2.4. The Measurements of PAPP-A Levels

Fasting blood samples were collected from the patient and control groups in the morning. Hemogram, CRP, and erythrocyte sedimentation rate (ECR) were studied. Blood samples collected for PAPP-A level were transferred into vacuumed tubes containing coagulation activator (BD Vacutainer®, Franklin Lakes, NJ, USA). After coagulation, blood samples were centrifuged at 1200 G for 15 min and stored at −80°C. PAPP-A levels of the thawed samples were studied according to the directions of the manufacturer on UniCel® DxC 600i (Beckman Coulter, Fullerton, CA) using enzyme-linked immunosorbent assay (ELISA) kits (detection range: 20 ng/ml–0.312 ng/ml, sensitivity: 0.06 ng/ml, intra-assay precision: <8%). All the samples were run in the same assay.

### 2.5. Statistics

Data obtained in the study were analyzed using SPSS 17.0 (SPSS for Windows, Chicago, USA) statistics program. The Kolmogorov–Smirnov test was used to evaluate whether the distribution of continuous variables was normal or not. Categorical data were given as frequency and percentage. Continuous variables were given as either (mean ± standard deviation) or as (median (minimum–maximum)) according to the results of the normality tests. A chi-square test was used for the comparison of categorical data. An independent sample *t*-test was used for the continuous variables with normal distribution. The Mann–Whitney *U* test was used for the continuous variables with abnormal distribution.

The Kruskal–Wallis test was used for the comparison of PAPP-A levels in OSAS patients categorized into 3 groups according to their AHI levels. Bonferroni correction was applied to compare two groups. For this result, *p* value below 0.05/3 = 0.017 was considered significant. Spearman correlation analysis was used for the correlations between continuous variables. For all other comparisons, *p* < 0.05 was accepted significant.

## 3. Results

The study comprised 44 patients with OSAS and 44 healthy control subjects. The age, gender, body mass index, and smoking ratio differences between the groups were statistically insignificant (Table 1). The ratios of snoring, apnea, daytime sleepiness, morning headache, morning fatigue, daytime fatigue, night sweating, and nocturia were 96%, 84%, 75%, 41%, 66%, 64%, 61%, and 55%, respectively. None of the control subjects had any of those complaints on a regular basis.

Patients and control subjects had similar hematologic parameters (Table 2). Both groups had similar respiratory function test results (Table 3).

Both groups had similar ESR and CRP. However, patients with OSAS had significantly higher PAPP-A levels (Table 4).

In OSAS patients, a significant positive correlation was detected between oxygen levels (minimum and mean) at night and PAPP-A levels. A significant negative correlation was detected between the AHI score and PAPP-A levels. No significant correlations were detected between the other parameters (BMI, NREM, Epworth score, ESR, CRP, and PAPP-A levels (Table 5).

A significant difference was detected in PAPP-A levels between the three groups categorized according to the AHI score. It was determined that the significant difference was caused by the higher PAPP-A levels in moderate severity group compared to low and high severity groups (Table 6). In terms of Epworth score, no significant differences were detected in PAPP-A levels between low and high score groups.

## 4. Discussion

In our study, we found that PAPP-A levels were significantly higher in OSAS patients compared to the control group. PAPP-A levels were significantly higher in patients with moderate severity group compared to other groups. While a positive correlation between minimum and mean oxygen levels at night and PAPP-A levels was observed, a negative correlation with AHI was detected.

In the first studies with PAPP-A, Bayes-Genis et al. reported that PAPP-A levels were significantly higher in patients with acute coronary syndrome [25]. Conti et al. reported that hypoxic, oxidative, and inflammatory stresses increased the bioactivity of PAPP-A and that the PAPP-A levels were correlated with myocardial damage [26]. Likewise, Eickhoff et al. and McAllister et al. also reported that risk factors like hypoxia and chronic inflammation are correlated with vascular endothelial dysfunction and atherosclerosis [27, 28].

Studies reported high PAPP-A levels in asthma, COPD, lung cancer, and chronic renal diseases where oxidative stress and inflammation play a role in pathogenesis [15, 17, 29]. Bulut et al. reported that PAPP-A levels were higher in lung cancer compared to the control group [15]. Similarly, Talay et al. showed that PAPP-A levels were significantly higher in patients with COPD compared to the control group [19]. Fialova et al. found a positive correlation between PAPP-A and CRP levels in patients who underwent dialysis [30]. In patients diagnosed with pulmonary embolism, it was reported that there were no correlations between the PAPP-A levels and inflammatory markers [16].

In a study, no correlation between CRP levels and PAPP-A levels was detected in COPD patients [19]. It is known that the increased sympathetic activity caused by hypoxia repeating through the night in OSAS leads to cardiovascular and metabolic changes. OSAS is among the known risk factors for ischemic heart disease, arrhythmia, hypertension, and metabolic syndrome. Many mechanisms like increased sympathetic activity, inflammation, endothelial dysfunction, metabolic disregulation, and oxidative stress play a role in OSAS pathogenesis. In the previous studies with OSAS patients, inflammatory cytokines like CRP, IL-6, and TNF*α* were reported to be correlated with the disease pathogenesis. A positive correlation was detected between IL-6 and isoprostane levels and the intensity of inhalation [31, 32]. In our study, while the PAPP-A levels were found higher in patients with OSAS compared to the control group, no differences were detected between CRP levels. No correlations were detected between PAPP-A levels and ESR and CRP levels. As far as we know, our study is the first study in the literature conducted about PAPP-A levels in OSAS.

In our study, surprisingly, a negative correlation was detected between PAPP-A levels and AHI. It was observed that PAPP-A levels significantly decreased as AHI moved above 30. In the meantime, a positive correlation was detected between minimum O_2_ and mean O_2_ levels at night and PAPP-A levels. In severe OSAS patients with significantly lower O_2_ levels, PAPP-A levels were also seen to decrease significantly. It made us think that the period when the PAPP-A levels decrease in severe OSAS may correspond to the period when the comorbidities are significant and when the compensatory mechanisms could not overcome oxidative stress and inflammatory activities. The comorbidites may be confounding factors in such a correlation between AHI/O_2_ levels and PAPP-A levels. However, a second possibility may be that the low number of participants included in our study may not be sufficient to explore the exact relationship between O_2_ levels and PAPP-A levels.

There were no correlations between PAPP-A levels and the duration of sleeping stages and Epworth sleep scale. In the light of these findings, high PAPP-A levels may assist in predicting the comorbidities which may occur in patients with OSAS, they may also be useful biomarkers in the management of patients with OSAS.

There are some limitations in our study. First of all, the number of patients is limited. Studies with larger cohorts may reveal the reasons for higher PAPP-A levels. Secondly, the distribution between patient groups while comparing the severity of disease was not equal. Thirdly, the study did not contain long-term follow-up data and PAPP-A data after treatment of the patients. Besides, there are no data to analyze the biopsy samples to exactly determine the role of PAPP-A levels in indicating especially the endothelial dysfunction in compensatory repair mechanisms.

## Figures and Tables

**Table 1:** Demographic and some clinical characteristics of the groups.

	OSAS (*n*=44)	Control (*n*=44)	*p*
Age (years)	44 ± 10	44 ± 12	0.791^*∗*^
Gender (male/female)	32/12	33/11	0.808^*∗*^
Body mass index (median (min–max))	29.41 **(23.89–40.44)**	28.58 **(22.42–45.70)**	0.166^*∗*^
Smoking (*n*, %)	18, 41%	23, 51%	0.549^†^

^*∗*^Mann–Whitney *U* test; ^†^chi-square test.

**Table 2:** Hematologic parameters of the groups.

	Control (*n*=44), median (min–max) or mean ± SD	OSAS (*n*=44), median (min–max) or mean ± SD	*p*
Leucocyte (K/ul)	6515 (3350–12800)	6885 (3460–10500)	0.981^†^
Lymphocyte (K/ul)	2145 ± 683	2267 ± 630	0.392^*∗*^
Neutrophils (K/ul)	3685 (1560–10200)	3895 (1410–7370)	0.893^†^
Eosinophils (K/ul)	147 (17–436)	178.5 (55–622)	0.124^†^
NLR	2.11 ± 0.97	1.86 ± 0.64	0.152^*∗*^
Haemoglobin (g/dl)	14.5 (12.1–17.3)	14.3 (10.3–17.1)	0.733^†^
Haematocrite (%)	43.1 (37.5–52.6)	44.1 (33.9–52.0)	0.935^†^
Mean corpuscular volume (fL)	91 (81–103)	91 (67.5–99.1)	0.823^†^
Red cell distribution width (%)	15.91 ± 0.98	16.11 ± 1.40	0.452^*∗*^
Mean platelet volume (fL)	7.86 ± 1.28	7.86 ± 1.02	0.994^*∗*^
Platelet distribution width	18 (16.5–20.8)	17.7 (16–22)	0.449^†^

^†^Mann–Whitney *U* test; ^*∗*^İndependent sample *t*-test.

**Table 3:** Respiratory function test results.

	Control (*n*=44) (mean ± SD)	OSAS (*n*=44) (mean ± SD)	*p*
Forced vital capacity (FVC) (L)	4.18 ± 0.99	4.21 ± 1.09	0.913
Forced vital capacity (FVC) (%)	101.32 ± 10.47	102.14 ± 13.52	0.757
Forced expiratory volume (FEV1) (L)	3.31 ± 0.79	3.34 ± 0.87	0.843
Forced expiratory volume (FEV1) (%)	96.93 ± 10.53	97.64 ± 13.18	0.78
Forced expiratory volume/forced vital capacity (FEV1/FVC)	79.14 ± 5.51	79.41 ± 5.38	0.819
Forced expiratory flow (FEF 25–75) (L/sn)	3.27 ± 1.09	3.40 ± 1.12	0.594
Forced expiratory flow (FEF 25–75) (%)	81.91 ± 22.43	84.61 ± 22.33	0.570
Peak expiratory flow (L)	6.64 ± 1.88	6.69 ± 1.78	0.902
Peak expiratory flow (%)	79.93 ± 16.18	81.05 ± 15.61	0.748

Independent sample *t*-test.

**Table 4:** Erythrocyte sedimentation rate, C reactive protein, and PAPP-A levels in the groups.

	Control (*n*=44) (median (min–max))	OSAS (*n*=44) (median (min–max))	*p*
Erythrocyte sedimentation rate (mm/sa)	9 (2–30)	9 (1–26)	0.517
C-reactive protein (mg/l)	1.55 (0–52.8)	2.10 (0–19.1)	0.509
PAPP-A (ng/ml)	0.971 (0.109–2.679)	2.350 (0.641–4.796)	<0.001

Mann–Whitney *U* test.

**Table 5:** The correlations between PAPP-A levels and clinical parameters.

Parameters	*r* ^*∗*^	*p*
Body mass index	0.126	0.241
Minimum O_2_ level in night	**0.309**	**0.042**
Mean O_2_ level in night	**0.407**	**0.006**
NREM1 (%)	0.136	0.377
NREM2 (%)	−0.143	0.356
NREM3 (%)	0.149	0.333
REM (%)	0.159	0.302
Epworth score	0.058	0.710
AHI score	−**0.460**	**0.002**
C-reactive protein	−0.002	0.988
Erythrocyte sedimentation rate	−0.088	0.415

^*∗*^Spearman correlation analysis.

**Table 6:** PAPP-A levels in groups organized according to AHI score and Epworth score.

Parameter	Groups	PAPP-A levels (median (min–max))	*p*
AHI score	Low (5–15)	1.26 ng/ml (0.64–2.99)	*p* < **0.001**, *p*^1^ and *p*^3^: **<0.001**, *p*^2^: 0.854
	Average (16–30)	4.26 ng/ml (2.75–4.79)	
	High (>30)	1.74 ng/ml (1.00–2.65)	

Epworth score	Low (<10)	1.89 ng/ml (0.64–4.79)	0.279
	High (≥10)	2.42 ng/ml (1.00–4.73)	

AHI: apnea-hypopnea score; *p*^1^: significance related to low-average groups; *p*^2^: significance related to low-high groups; *p*^3^: significance related to average-high groups.

## Data Availability

The data used to support the findings of this study are available from the corresponding author upon request.
